# First reported case of unrepaired tetralogy of Fallot complicated with coronavirus disease-19 (COVID-19)

**DOI:** 10.1017/S1047951120001821

**Published:** 2020-06-11

**Authors:** Mansoor Moazenzadeh, Fatemeh Jafari, Mehrdad Farrokhnia, Maryam Aliramezany

**Affiliations:** 1 Cardiovascular Research Center, Institute of Basic and Clinical Physiology Sciences, Kerman University of Medical Sciences, Kerman, Iran; 2 Infectious Diseases Department, Afzalipoor Hospital, Kerman, Iran

**Keywords:** Adult CHD, SARS-CoV-2, tetralogy of Fallot

## Abstract

The incidence of novel coronavirus disease-19 (nCoV-19) and its associated complications is higher in high-risk groups. In this article, we explain the symptoms and course of the disease and the treatment for an adult patient with CHD who has been infected with novel nCoV-19.

The new coronavirus disease-19 (nCoV-19) which began in Wuhan, China, in December 2019, has dramatically spread to several countries around the world.^1^ Although the prevalence of the disease has not yet been fully established and numerous articles have been reported in recent weeks about the disease, there is still little evidence of the course of the disease among adult patients with CHD.^2^

Therefore, herein we aimed to describe first reported case of adult patient with CHD who was infected with novel SARS-CoV-2.

## Case report

A 48-year-old man with past history of CHD, who has been treated with O2 at home for several years because of dyspnoea and cyanosis, was admitted to our hospital on 8 April, 2020, due to exacerbation of dyspnoea and central cyanosis for 2 days. During physical examination, we heard harsh systolic murmur in upper left sternal border and generalised coarse crackle in both lungs. Mild clubbing was visible on examination of the limbs. His habitual history indicated that the patient was a cigarette smoker. We noticed a history of diagnosed but untreated CHD because of inability to afford treatment expenditures at the time of diagnosis.

**Figure 1.**
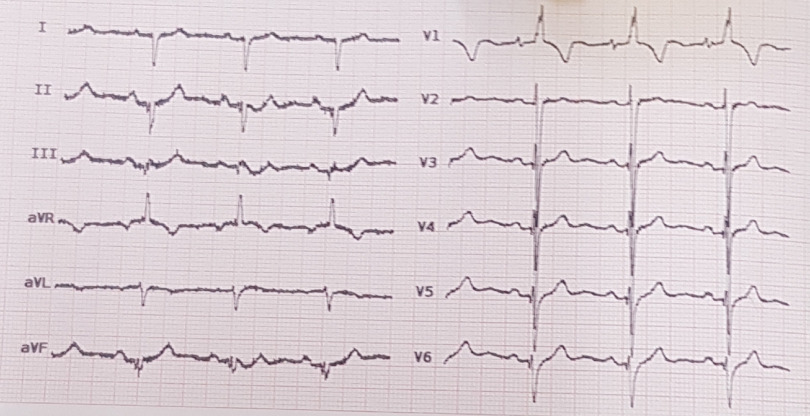
ECG shows tall R waves in V1; prominent P waves in V1; undetermined axis.

At admission, the patient’s vital signs were as follows: BP:130/80 mmHg, PR:86 beats per minute, RR:23 per minute, T:39.5, and SPO2:70% in room and 86% by nasal cannula. First VBG was PH:7.45, PCO2:47, HCO3:33, and second VBG after initial treatment with O2 and inhaler was PH:7.47, PCO2:37, and HCO3:27. Electro Cardio Gram (ECG) showed increased right ventricular forces (tall R waves in V1); right atrial enlargement (prominent P waves in V1); right ventricular hypertrophy; and undetermined axis (Fig 1). Other initial laboratory findings were WBC:11,100 (per μl), Hb:18.2 (g/dl), PLT:195,000 (per μl), lymphocyte count: 968 (per μl), CRP:52 (mg/L), LDH:542U/L, and procalcitonin (PCT):0.7 ng/ml (normal range: less than 0.5 ng/ml). Liver and kidney tests were normal.

**Figure 2.**
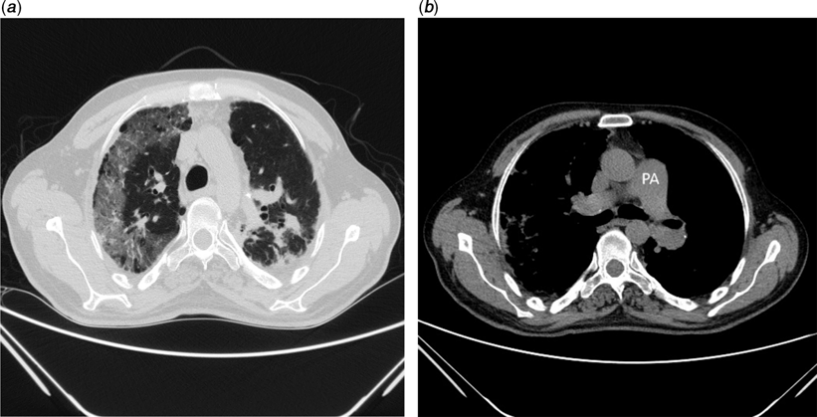
Chest CT scan shows (***a***) ground-glass appearance and peripheral round opacity; (***b***) dilation of pulmonary artery.

After that, chest X-Ray was taken and with regard to patchy infiltration, he was susceptible to COVID-19 infection. Hence, chest CT-scan and Polymerase Chain Reaction (PCR) were requested. Chest CT-scan revealed bilateral lung involvement, ground glass appearance, and peripheral round opacity all in favour of COVID-19. Furthermore, distinct cardiomegaly and dilation of pulmonary trunk were observed (Fig 2 a,b). Moreover, patient’s nasopharyngeal swab tested positive for 2019-nCoV PCR assay. Patient was managed by a team of physicians including fellowship of adult CHD, anesthesiologist, and infectious diseases specialist.

**Figure 3.**
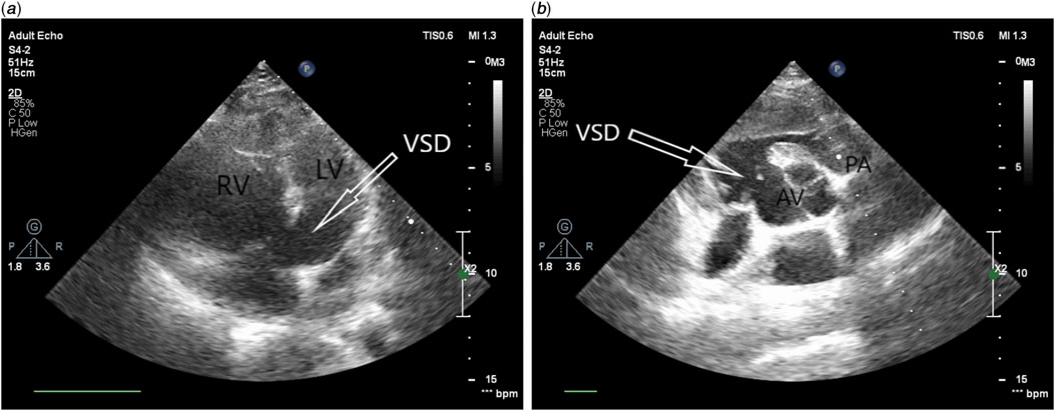
Doppler echocardiography: (***a***) in 4C view shows large sub-aortic VSD with extension to inlet; (***b***) in SAX shows large VSD; VSD: ventricular septal defect; PA: pulmonary artery; AV: aortic valve.

Patient received high-flow oxygen and common antiviral protocol of COVID-19. In addition to high level of procalcitonin and suspected concurrent bacterial pneumonia, he was also treated by an infectious disease specialist with antibiotics. Despite mentioned treatments, his dyspnoea progressed and SO2 decreased to 68% in the second day of hospitalisation, and patient was referred to ICU where he received prophylactic heparin to prevent thromboembolism, but due to absence of severe respiratory distress, we decided not to intubate him. On the third day of hospitalisation, due to the lack of appropriate therapeutic response and the disproportionate low level of oxygen saturation, limited portable echocardiography was performed and showed: situs solitus; D-looped ventricle; normal continuity of inferior vena cava to right atrium; moderate left ventricle enlargement and systolic dysfunction, severe right ventricle enlargement and systolic dysfunction and severe right ventricular hypertrophy; large sub-aortic ventricular septal defect (3.2 cm) with extension to inlet and bidirectional shunt (predominantly right to left); overriding of aorta; pulmonary artery originate from right ventricle with thick and dome-shaped pulmonic valve; relative dilation of pulmonary trunk (3.9 cm) and acceptable pulmonary branches (Fig 3). All of these findings were highly suggestive of tetralogy of Fallot anomaly.

Consequently, with regard to these findings, we added diuretic and angiotensin-converting enzyme inhibitors to his medical regimen with adequate dosage which led to partially improved condition and increased blood oxygen saturation (89%). Fourteen days after the start of treatment, due to the relative improvement of the repeated chest CT-scan findings and his general condition, the patient was discharged with the necessary medication instructions and recommendations. Furthermore, we advised him to undergo a complete follow-up echocardiography and the necessary diagnostic and therapeutic measures.

## Discussion

This is the first reported case of 2019-nCoV in Adult Congenital Heart Disease (ACHD) patients which illustrates several aspects of this emerging outbreak that are not fully understood yet, including the full spectrum of clinical illness. At present, our information about the clinical presentation and course of COVID-19 is limited, but numerous clinical complications such as severe pneumonia, respiratory failure, acute respiratory distress syndrome, and cardiac injury have been mentioned in studies reported from China.^3–5^

The chief complaint of our patient was exacerbation of shortness of breath irresponsive to oxygen therapy. In contrast, recent studies have shown that the most common symptoms of COVID-19 patients are fever and dry cough, indicating that the progress and the nature of the disease are different in various patients. The most common laboratory disorder reported in COVID-19 patients is lymphocytopenia (in 83.2% of the patients on admission)^2^ that was also observed in our patient, which then gradually improved during treatment. The radiological findings in the patient’s CT scan are similar to those mentioned in previous studies on the disease (pattern of ground-glass and consolidative pulmonary opacities, often with a bilateral and peripheral lung distribution);^6,7^ however, the apparent cardiomyopathy, thick and dome-shaped pulmonary valve, and relative dilatation of the pulmonary trunk are specific to the underlying disease. With this in mind, it is important to consider other specific signs when examining patients’ para-clinical findings of corona patients, as this may affect the course of treatment and the need for further diagnostic methods. Limited echocardiography indicated abnormal pulmonary valve with mildly increased gradient, highly suggestive of spectrum of tetralogy of Fallot anomaly.^8^

Furthermore, polycythemia is a common laboratory finding in cyanotic patients with CHD, and in some situations, phlebotomy is performed based on ferritin level, hematocrit level, and patient’s symptoms. But for the reported patient, we did not prescribe phlebotomy because of insufficient evidence in acute phase of COVID-19.

Since no study on the treatment of the virus has been reported in adult patients with CHD, we decided to treat this case based on the protocols which have been proposed to treat adult patients with cardiovascular disease.^1^ Furthermore, in patient with cyanotic heart disease and non-restrictive ventricle septal defect, right ventricle blood passes to left systemic circulation and exacerbates hypoxia. Hence, any manoeuvres, which decrease amount of the right to left shunt, improve hypoxia.^9^ Therefore, we decided to start the specific treatments of patients with ventricular dysfunction^10^to decrease cardiac afterload and filling pressure. Regarding the different physiology and anatomy of adult patients with CHDs, efforts are necessary to be made by related national and international societies of adult CHDs to collect data from a number of assumed and established cases to better recognise the prognosis of this special group and to develop specific protocols for treating adult CHDs patients who are infected by COVID-19.^1^

## Conclusion

To sum up, the reported case highlights the need to determine the full spectrum and natural history of disease, pathogenesis, and duration of viral shedding associated with 2019-nCoV infection to inform clinical management and public. Additionally, adult patients with CHD who are infected by COVID-19 seem to benefit from their own specific treatments as well as routine antiviral therapies for the infection. Furthermore, we advised in patients with unusual presentation or unfavourable response to treatment, another para-clinical study was done.

## Availability of data and material

Data sharing is not applicable to this article as no datasets were generated or analysed during the current study.
